# Effects of rhBMP-2 Loaded Titanium Reinforced Collagen Membranes on Horizontal Bone Augmentation in Dogs

**DOI:** 10.1155/2017/7141296

**Published:** 2017-10-17

**Authors:** Ki-Sun Lee, Yu-Sung Jeon, Sang-Wan Shin, Jeong-Yol Lee

**Affiliations:** ^1^Department of Prosthodontics, Korea University Guro Hospital, Seoul, Republic of Korea; ^2^Graduate School of Clinical Dentistry, Korea University, Seoul, Republic of Korea

## Abstract

The aim of this study was to evaluate the efficacy of growth factor loaded collagen membranes on new bone formation during horizontal bone augmentation. Mandibular defects (4 × 4 × 4 mm) were surgically prepared in six male beagle dogs, which were then protected with one of three types of membranes: (1) titanium mesh, (2) titanium reinforced collagen, or (3) rhBMP-2 loaded titanium reinforced collagen. Animals were euthanized 8 and 16 weeks after surgery, and nondecalcified specimens were prepared and histomorphologically investigated to determine the degree of osteogenesis. Data were analyzed with Friedman test. With respect to the degree of osteogenesis at earlier stage (8 weeks after surgery), there was significantly higher new bone ratio in rhBMP-2 loaded membrane group (*p* > 0.05). However, with respect to the long-term results (16 weeks after surgery), there were no significant differences among the three membranes (*p* > 0.05). Based on histomorphometric analysis, there were no significant differences in horizontal bone gaining ratio (*p* > 0.05).

## 1. Introduction

Bone resorption following tooth extraction or periodontal disease can result in both horizontal and vertical bone loss, which in turn can complicate implant installation in the area. A minimum amount of bone is essential for the long-term success of implant restorations [1, 2], and several methods have been introduced to compensate for the lack of bone during implant installation. Three factors are required for ideal bone formation. First, osteoinductive growth factors are needed as signaling molecules to induce differentiation. Second, an osteoconductive matrix is needed to serve as a scaffold to offer advantageous conditions for cell proliferation and differentiation. Third, osteoprogenitor cells are needed to function as signal receptors [3]. Taking these factors into consideration, various efforts have been made to increase the amount of bone using different combinations of membranes, growth factors, and bone graft materials.

Inducing bone regeneration with barrier membranes has been attempted clinically since the early 1980s, where different materials have been developed to facilitate cell attachment, increase cell proliferation, and enhance the effects of membranes for promoting cell movement. Barrier membranes create space without causing connective tissue and cell infiltration in the defect area, thereby promoting new bone formation [4–6]. When performing GBR (guided bone regeneration), the amount of bone regenerated below the membrane is directly related to the space below the membrane [7]. However, membrane collapse can lead to space reduction, as the membrane alone has a limited ability to act as a scaffold in the setting of soft tissue compression. In order to address this problem, titanium mesh has been introduced as an effective scaffold material for maintaining space [8–10].

Bone morphogenetic proteins (BMPs) stimulate mesenchymal cells and increase bone collagen synthesis to promote endochondral bone formation. Among the known BMPs, rhBMP-2 is the most effective osteogenic factor for inducing early formation of trabecular woven bone as well as lamellar bone remodeling [11]. Although BMPs are the most important growth factors for bone formation and bone healing, they have a short half-life and break down rapidly when injected directly into an area of bony defect. As a result, the use of BMP is currently limited clinically, as a proper delivery system is required to prolong its effects during the healing period, which lasts several months [12–14]. However, a number of delivery systems have been investigated to overcome the short half-life of BMPs, with the main function of these delivery systems being to maintain growth factors to allow sufficient time for cell proliferation and differentiation [15, 16].

Considering the factors that promote bone formation described above, ideal conditions for bone formation include a proper delivery system to maintain space in the defect area, prevent infiltration of connective tissue, and prolong the effect of growth factors. In the present study, we investigated the effects of titanium reinforced collagen membranes as a carrier of rhBMP-2 on horizontal bone augmentation in mandibular premolars of dogs.

## 2. Materials and Methods

### 2.1. Animals

Six 2-year-old beagle dogs with an average weight of 15 kg were used in this study. Animal selection and management and the surgical protocol were approved by the Ethics Committee on Animal Experimentation at Chonnam National University (CNU IACUC-YB-R-2010-10). All animals were assessed and cared for by a veterinarian under standard laboratory conditions.

### 2.2. Surgery and Postoperative Care

**Figure 1:**
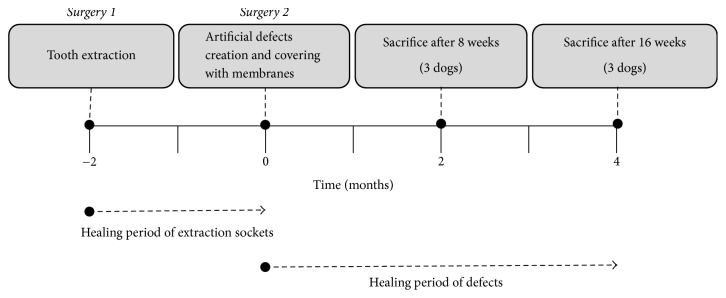
Study design and schedule.

**Figure 2:**
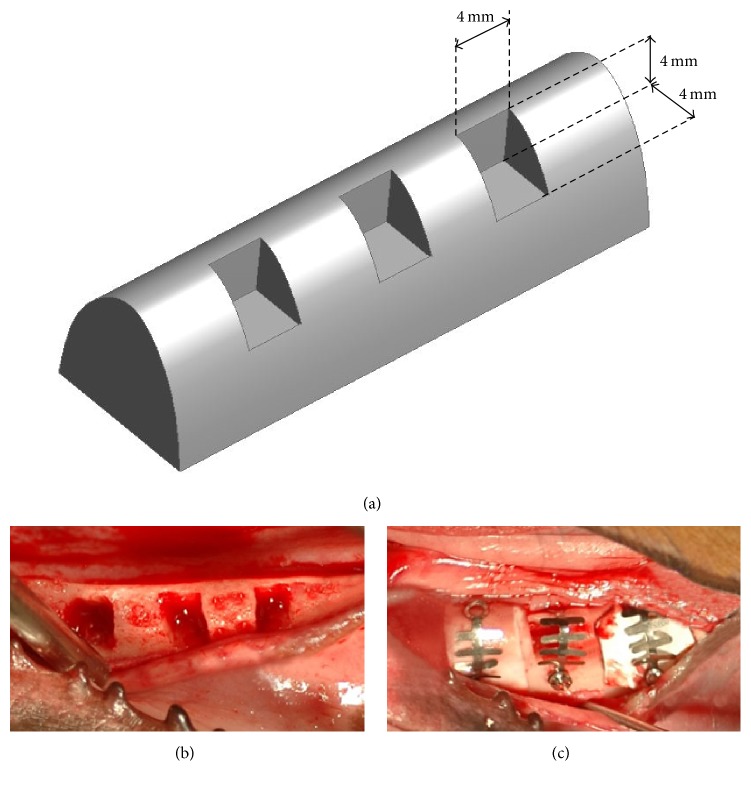
(a) Design concept of the defects. (b) Defect formation. (c) Coverage of defects with randomly assigned membranes.

**Figure 3:**
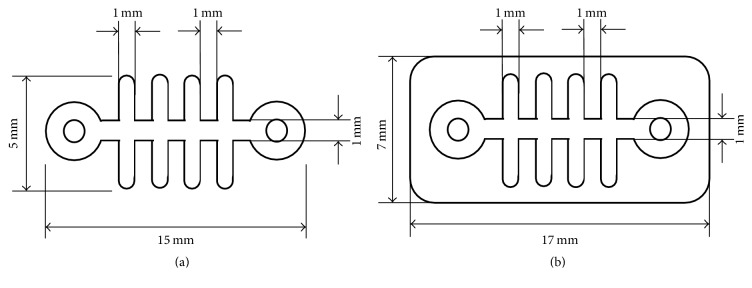
Membrane design schematics (a) Titanium mesh. (b) Titanium reinforced collagen membrane.

The overall study design and surgery protocol are shown in Figure 1. All surgical procedures were performed under general anesthesia, and local infiltration anesthesia was used at the surgical sites. Initially, all premolars and first molars were bilaterally extracted. After an eight-week healing period following the initial surgery, a second surgery for defect formation and membrane protection was performed. Specifically, three box-type lateral bone defects (length: 4 mm, height: 4 mm, and depth: 4 mm) were created on each side of the mandible as shown in Figure 2. Immediately after the second surgery the defects were protected using one of three membranes that were assigned at random (Table 1). The membranes were custom designed and fabricated for this study (Figure 3). To fabricate the rhBMP-2 loaded membrane, 25 *μ*g of rhBMP-2 (Cellumed, Seoul, Korea) was mixed with 1 cc of saline to a concentration of 25 *μ*g/ml, and 0.5 cc of the solution was soaked on the collagen membrane. Titanium pins (Dentium Co., Seoul, Korea) were used for membrane fixation to enhance stability. The flaps were then carefully released and sutured with 4-0 Vicryl® (Johnson and Johnson, New Brunswick, NJ, USA). Antibiotics were administered immediately after the second surgery and again after 48 h. All animals were maintained on a soft diet and plaque formation was controlled by daily flushing of the oral cavity with a 2% chlorhexidine solution until euthanasia.

### 2.3. Sacrifice and Specimen Preparation

Three dogs were euthanized 8 weeks after the second surgery, and the remaining three dogs were euthanized after 16 weeks. Euthanasia was performed with an overdose of pentobarbital sodium delivered intravenously. The mandibles of the euthanized dogs were block-resected and the segments were immersed in a fixing solution. Next, the prepared block specimens were placed in a matrix consisting of methyl methacrylate resin and polymerized at room temperature. The resin-polymerized specimens were vertically cut into slices 0.5 mm thick with a diamond blade, and the specimens were further processed with petrographic grinding to yield final specimens with a thickness of 30–50 *μ*m. The resulting specimens were then decalcified with EDTA and stained with hematoxylin-eosin.

### 2.4. Histomorphometric Analysis

**Figure 4:**
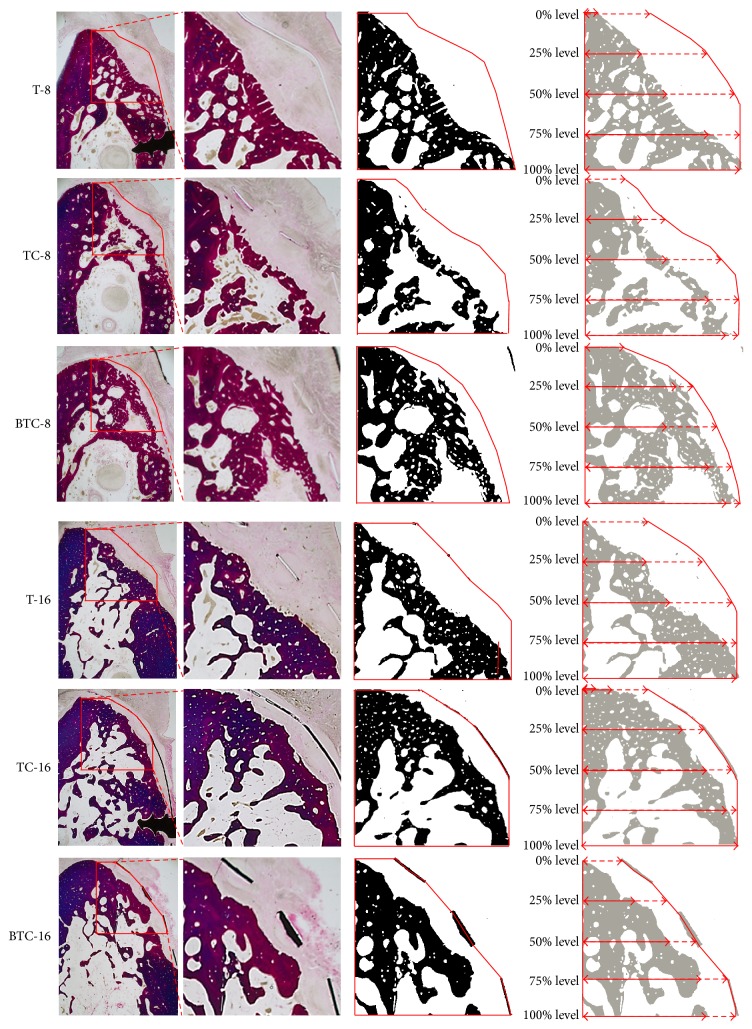
Histomorphometric analysis. Red polygonal areas represent AOI boundaries. New bone is represented in black in the third column. Measurements of horizontal bone growth at different defect heights are shown in the last image column.

To observe bone regeneration, images of specimens were captured using a Leitz DM-RBE® light microscope (Leica, Wetzlar, Germany) equipped with a D90 digital camera (Nikon, Tokyo, Japan). Histomorphometric measurements were performed using ImageJ (National Institutes of Health, Bethesda, MD, USA). The area of interest (AOI) was defined by the boundary of a fan-shaped polygonal defect inside the membrane (height: 4 mm, depth: 4 mm; Figure 4). The following parameters were calculated within AOIs:

- New bone ratio (NBR; %): area occupied by new bone/AOI × 100 (%)
- Horizontal bone gain ratio at different defect levels (BGR; %): at height levels of 25%, 50%, 75%, and 100% of the former bone defect, distance from the lingual boundary of the AOI to the most buccal aspect of newly formed bone/the distance from the lingual boundary of the AOI to the buccal boundary of the AOI.

### 2.5. Statistical Analysis

All experimental data are expressed as the mean with standard deviation. All statistical analyses were performed using SPSS ver. 22 (IBM, Armonk, NY, USA). Due to the limited sample size, comparisons between experimental groups were made using Friedman test. Post hoc analysis was performed using Bonferroni correction and the significance level was set at 0.05.

## 3. Results

**Figure 5:**
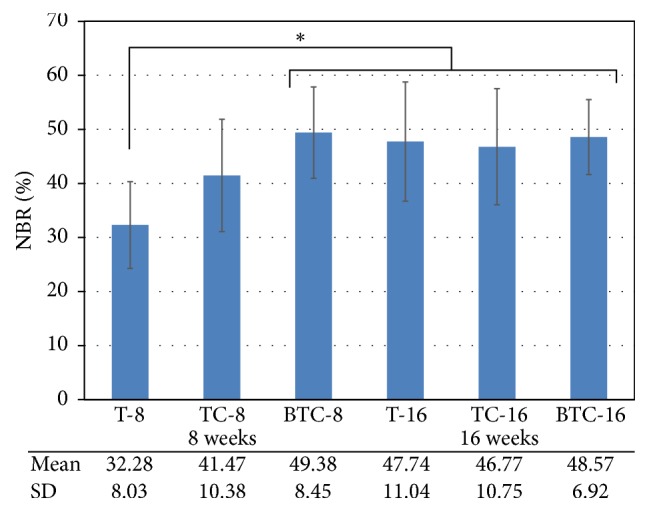
Mean and standard deviation of the new bone compartment ratio (NBR; %) within membrane-protected bone defects (*n* = 6; %). The asterisk indicates statistically significant differences (*p* < 0.05).

**Figure 6:**
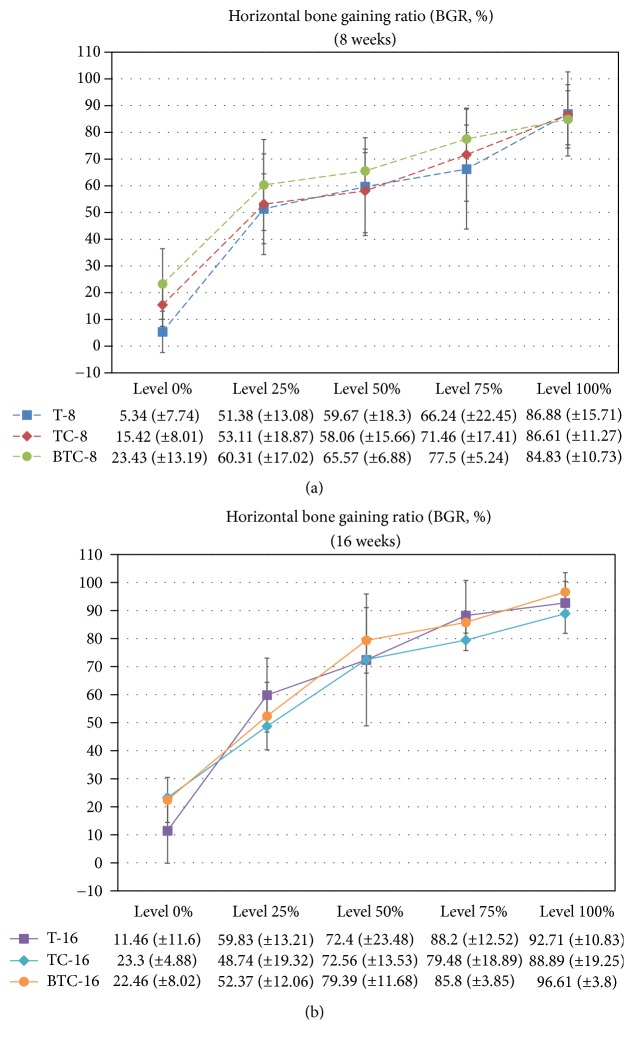
Mean and standard deviation of the horizontal bone gaining ratio (BGR; %) according to defect height level at 8 and 16 weeks.

The results of our histomorphometric analysis are summarized in Figures 5 and 6. The percentages of new bone compartments (NBR; %) within the membrane-protected bone defects were compared according to each membrane type for each period (Figure 5). The experimental groups at 16 weeks and rhBMP-2 loaded titanium reinforced collagen membrane group (BTC-8) at 8 weeks had significantly higher NBR% values compared to the titanium membrane group at 8 weeks (T-8). With respect to horizontal bone growth measurements of ridge augmentation (Figure 6), there were no significant differences between experimental groups according to time, type of membrane, and height level. However, the lowest value tendency of BGR% was found at the 0% height level for the titanium membrane group for both periods.

## 4. Discussion

The principles of GBR are to prevent soft tissue cell infiltration in the area of the defect and to promote specific activation of osteogenic cells [17]. A barrier membrane is used to maintain the space in the defect area, which promotes osteogenesis by stabilizing the initial blood clot and blocking soft tissue infiltration [18]. Titanium mesh, which was used to maintain space in this study, has excellent space ability to form space. Specifically, it prevents soft tissue collapse and compression during the bone healing period, thereby ensuring that bone substitutes remain below the membrane.

The aims of this study were to evaluate the effect of rhBMP-2 loaded titanium reinforced collagen membranes on new bone formation in a horizontal bone defect area. RhBMP-2 has strong osteoinductive properties in bone regeneration [19, 20]; however, it also has a short half-life in vivo due to rapid degradation. For this reason, we used a collagen membrane as a carrier of rhBMP-2. Consistent with this approach, McKay et al. [21] showed that an absorbable collagen sponge (ACS) and Infuse Bone Graft® mixed with 1.5 mg/cc of rhBMP-2 for sinus elevation and alveolar ridge augmentation are effective in promoting osteogenesis. In addition, Chang et al. [22] compared the use of a collagen membrane as a carrier of rhBMP-2 with a collagen membrane used to cover a collagen matrix and rhBMP-2 incorporated bovine hydroxyapatite. They did not identify a significant difference between the two approaches and suggested that use of a collagen membrane loaded with rhBMP-2 is a useful treatment approach. Similarly, Fiorellini et al. [23] showed that the combination of rhBMP-2 and a collagen sponge has a satisfactory effect on bone formation, while Miron et al. [24] reported that a collagen membrane loaded with BMP-2 has a noticeable effect on adhesion, proliferation, and differentiation of osteoblasts.

The effectiveness of rhBMP-2 has been verified in many studies, although the reported results vary according to treatment applications, conditions, and methods of evaluation. In the present study, a titanium mesh-reinforced collagen membrane was used to maintain space in the defect area, stabilize blood clots, and prevent soft tissue infiltration. The present study was designed to exclude the effects of bone substitutes in order to identify the effect of rhBMP-2 alone. Specifically, we compared the effects of three different type membranes, namely, titanium mesh alone, titanium reinforced collagen membrane, and rhBMP-2 loaded titanium reinforced collagen membrane. Thus, this experiment was assumed to be performed under the same space maintenance conditions, without additional membrane, with collagen membrane, and with rhBMP-2 loaded collagen membrane, respectively. Each material's effect on osteogenesis was analyzed to identify the ideal conditions for promoting bone formation in the same space maintenance condition. The initial defect areas had dimensions of 4 × 4 × 4 mm and were used to simulate dehiscence or fenestration defect, the most common forms of defects used in previous studies. All membranes were fixed with a tag to minimize membrane movement.

With respect to NBR% within defects, we found no statistically significant differences between the three membrane groups at the 16-week time point. However, from a short-term perspective (8 weeks), rhBMP-2 loaded titanium mesh-reinforced collagen membranes had a significantly higher NBR% compared to the group treated with titanium mesh alone. These results may have been due to the favorable effect of rhBMP-2 on osteoinduction. Zellin and Linde [25] showed that a barrier membrane is crucial for promoting bone formation below the membrane but at the same time interrupts the osteoinductive capacity of BMP and the migration of macrophages and inflammatory cells. Thus, an rhBMP-2 loaded membrane generates greater bone formation compared to a barrier membrane lacking rhBMP-2. Consistently, a small amount of bone formation adjacent to the barrier membrane was observed when rhBMP-2 was used with a collagen carrier.

Although not statistically significant, we observed a greater amount of new bone formation adjacent to rhBMP-2 loaded barrier membranes, as well as an increased BGR% compared to the other groups at the earlier stage of osteogenesis (8 weeks). However, use of the rhBMP-2 loaded barrier membrane did not affect overall bone morphology at the longer time point of this study. These results may have been due to the effect of the space maintaining capacity of the barrier membranes such as titanium mesh, which is an important factor for promoting overall bone morphology. Additional studies with more experimental groups will be needed to fully determine the relative importance of the various membrane components evaluated in this study.

## 5. Conclusions

In the present study, we sought to verify the efficacy of an rhBMP-2 loaded membrane on osteogenesis. To this end, present study employed three types of experimental membranes: titanium mesh alone, a titanium reinforced collagen membrane, and an rhBMP-2 loaded titanium reinforced collagen membrane. Within the limitations of this study, with respect to new bone formation ratio, the use of rhBMP-2 loaded collagen membrane had a significant effect on osteogenesis at earlier stage of osteogenesis (8 weeks after surgery) compared to the other types of membranes that were investigated. However, all experimental groups exhibited similar success with respect to new bone formation from a longer-term perspective (16 weeks after surgery).

## Figures and Tables

**Table 1:** Experimental group design.

Groups	Titanium mesh only	Titanium reinforced collagen membrane	rhBMP-2 loaded titanium reinforced collagen membrane
8 weeks	T-8	TC-8	BTC-8
16 weeks	T-16	TC-16	BTC-16
