# Corrigendum to “Effects of rhBMP-2 Loaded Titanium Reinforced Collagen Membranes on Horizontal Bone Augmentation in Dogs”

**DOI:** 10.1155/2018/8318136

**Published:** 2018-03-20

**Authors:** Ki-Sun Lee, Yu-Sung Jeon, Sang-Wan Shin, Jeong-Yol Lee

**Affiliations:** ^1^Department of Prosthodontics, Korea University Guro Hospital, Seoul, Republic of Korea; ^2^Graduate School of Clinical Dentistry, Korea University, Seoul, Republic of Korea

In the article titled “Effects of rhBMP-2 Loaded Titanium Reinforced Collagen Membranes on Horizontal Bone Augmentation in Dogs” [1], the Authors' Contributions section was missing and should be added as follows:
